# Evaluation of the Phenolic Components, Fiber Content, Antioxidant Activity, and Prebiotic Capacity of a Shortbread Cookie Fortified with Hazelnut Skin Waste

**DOI:** 10.3390/foods13233814

**Published:** 2024-11-26

**Authors:** Lara Costantini, Giacomo Di Matteo, Martina Felli, Daniel V. Savatin, Luisa Mannina, Nicolò Merendino

**Affiliations:** 1 Department of Ecological and Biological Sciences (DEB), Tuscia University, Largo dell’Università snc, 01100 Viterbo, Italy; 2 Department of Chemistry and Technology of Drugs, Sapienza University of Rome, Piazzale Aldo Moro 5, 00185 Rome, Italy; 3 Department of Agriculture and Forestry Science (DAFNE), Tuscia University, Via S. Camillo de Lellis snc, 01100 Viterbo, Italy

**Keywords:** hazelnut by-product, hazelnut cuticle, hazelnut pellicle, Tonda Gentile Romana hazelnut, food reformulation, phenolic compounds, antioxidant activity, prebiotic activity

## Abstract

Food reformulation is a strategy to make healthier foods by using food waste matrices that are still nutritionally valid. A shortbread cookie was reformulated replacing hazelnut skin (HS) of the Tonda Gentile Romana variety (5% and 10%) to refined flour and proportionally decreasing the butter amount. This resulted in significant, two- and five-fold, increases in the antioxidant capacity compared with the control, in the 5% and 10% fortified recipes, respectively. Among the most important antioxidants, gallic acid, catechin, phloridzin, and protocatechuic acid were found. Moreover, here we found, for the first time, that HS from the Romana variety had a high total fiber content (44.13 g/100 g), most of which was insoluble fiber. Therefore, HS 10% addition to the shortbread cookie recipe caused a significant increase in fiber content, making the experimental cookie earn the nutritional claim of “high fiber content”. Finally, preliminary evidence demonstrated that 10% HS, in comparison to 5%, following in vitro upper gastrointestinal digestion, conferred significant prebiotic activity in an in vitro culture of *L. rhamnosus*. Therefore, from the perspective of the circular economy, HS could be a valuable ingredient to increase the antioxidant and prebiotic activities of conventional foods.

## 1. Introduction

Recent reports showed a continuous decrease in adherence to the Mediterranean diet, even in those countries where this dietary pattern was born [1]. Among the most poorly followed guidelines is the consumption of fruit and vegetables, which correlates with an overall daily intake of fibers significantly lower than those suggested [2,3]. Indeed, the European Food Safety Authority (EFSA) recommends a daily fiber consumption of at least 25 g/day for adults to prevent non-communicable diseases, overweight, and obesity [4]. However, European fiber consumption does not exceed 18 g/day [3]. The importance of fiber consumption also lies in fiber’s prebiotic role, which is useful for maintaining the health of the intestinal microbiota. The lack of a healthy microbiota, called dysbiosis, is correlated with the onset of different pathologies, such as colorectal cancer. Over time the concept of prebiotics has evolved, and while waiting for further evidence, polyphenols are now considered prebiotic candidates [5]. In addition to the reduction in fiber intake from the diet, in Europe there is significant consumption of ultra-processed foods, especially fine bakery products rich in saturated fatty acids and simple sugars, which can significantly worsen health status [6]. For this reason, many countries and institutions have adopted policies to promote food reformulation using healthier ingredients and to reduce the risk of diet-related non-communicable diseases [7]. Recently, food matrices discarded by the agri-food industries during the production phase have also been considered to be healthy ingredients for food reformulation due to their high content of important nutritional components, especially fiber and antioxidants, and with the dual purpose of reducing global food waste in the context of the circular economy. Among these, hazelnut skin (HS) by-product is attracting considerable interest for its high antioxidant capacity (about 42 times higher than the hazelnut fruit [8,9]), high fiber content (50–70%) (even higher than conventional sources such as whole wheat flour that contain 10–15% of fibers [10]), and high content of tocopherols and oleic acid [11]. Beyond nutritional features, preliminary evidence shows that HS also has interesting sensory properties, which, although different from the whole hazelnut [12], are appreciated by potential consumers [13]. In the literature, HS has already been used as an ingredient to fortify burgers, yogurts, chocolate spreads, and bakery products [14]. Shortbread cookies are fine baked goods that have a large worldwide market, but their nutritional composition does not meet nutritional guidelines, because of the high amount of saturated fatty acids and simple sugars and the low fiber content. For this reason, many efforts are being made today to reformulate this product to make it healthier, also by introducing innovative ingredients. In this context, HS from the Romana hazelnut variety could be an interesting solution. Indeed, we previously reformulated a classic shortbread cookie recipe replacing refined wheat flour with HS powder of the Tonda Gentile Romana hazelnut variety (HSR; 5% and 10%). Moreover, the amount of butter in the recipe was proportionally decreased, considering the amount of lipids provided by HS powder, inducing an increase in the unsaturated fatty acid (UFA) to saturated fatty acid (SFA) ratio from 0.45, in the classic shortbread recipe, to 0.60 in the 10% HS experimental recipe, with a 20% increase in the monounsaturated oleic acid content and a 15.7% decrease in the saturated palmitic acid content, in addition to a significant ash increase and good consumer acceptance rates from sensory evaluation [13]. Here, additional data are shown by determining the phenolic components, total fiber content, antioxidant activity, and prebiotic capacity of *L. rhamnosus* following in vitro digestion of the experimental cookies.

## 2. Materials and Methods

### 2.1. Hazelnut Skins, Shortbread Cookies, and Chemicals

Hazelnut skins of the Tonda Gentile Romana (R), Tonda di Giffoni (G), Tonda Gentile delle Langhe (L), and Turkish Tombul (T) varieties were obtained from a local producer (Bionocciola srl, Carbognano, Viterbo, Italy) after roasting hazelnuts of each variety at 150 °C for 24 min and storing in vacuum-packed plastic bags at 4 °C in the dark. Shortbread cookies were produced as indicated in our previous publication [13], containing 5% (HSc5%) and 10% (HSc10%) of hazelnut skin powder; HSR was chosen as the ingredient for cookie fortification based on the best nutritional content [13]. A classic shortbread cookie recipe (CTRc; control cookie) was used as a control (classic recipe: 48.3% wheat flour, 20.2% butter, 19% sugar, 11.6% eggs, 0.1% vanillin, 0.8% baking powder; experimental recipes: 5% and 10% HSR replaced to wheat flour) [13]. All samples (i.e., hazelnut skins and cookies) were ground in a laboratory mill (IKA^®^ A11 basic Analytical mill (IKA^®^-Werke GmbH & CO., KG, Staufen im Breisgau, Germany) and then stored at −80 °C until further processing. All chemicals were purchased from Merck KGaA (Darmstadt, Germany) unless otherwise specified.

### 2.2. Extracts’ Preparation

For analysis of the total polyphenol content (TPC), total flavonoid content (TFC), total antioxidant capacity (TAC), and quantitative polyphenol determination by RP-HPLC, all samples were extracted according to Costantini et al., 2014 [15]; three biological and two technical replicates were processed and analyzed. Briefly, experimental powders of both hazelnut skins and cookies were extracted overnight in the dark with 80% aqueous methanol (1:25, *w*/*v*) for complete extraction of the antioxidant components. Then, the samples were centrifuged at 10,000 rpm (ALC PK121R centrifuge; Bodanchimica s.r.l., Cagliari, Italy) for 10 min at 4 °C. The supernatants were collected and stored at −80 °C until further processing.

### 2.3. TPC, TFC, and TAC

TPC, TFC, and TAC were analyzed for hazelnut skin of different varieties and experimental cookie samples, as described in Farinon et al., 2024 [16]. Briefly, TPC was determined using the Folin–Ciocalteu standard method: 30 μL of deionized water was added to 10 μL of methanolic extract, 10 μL of Folin–Ciocalteu reagent, and 200 μL of 30% Na_2_CO_3_. After 30 min at room temperature (RT), the absorbance of the mixture was measured at 725 nm. TFC was determined using the aluminum chloride colorimetric method: the extracts (50 μL) were mixed with 130 μL of 95% ethanol, 10 μL of 10% aluminum chloride hexahydrate (AlCl_3_), and 10 μL of 1 M potassium acetate (CH_3_COOK). After incubation at RT for 30 min, the absorbance of the reaction mixture was measured at 415 nm. TAC was assessed by ferric reducing antioxidant power (FRAP) and 2,2′-azino-bis (3-ethyl-benzothiazoline-6-sulfonic acid) (ABTS^•+^) radical scavenging activity assays. For FRAP, 160 μL of FRAP assay solution (20 mM ferric chloride solution, 10 mM TPTZ solution, and 0.3 M acetate buffer) was mixed with 10 μL of the sample, standard, or blank and dispensed into each well of a 96-well plate; the absorbance was measured at 595 nm at 37 °C after 30 min of incubation. ABTS^•+^ was evaluated using an OxiSelect™ Trolox Equivalent Antioxidant Capacity (TEAC) Assay Kit (ABTS) (Cell Biolabs Inc., San Diego, CA, USA) following the manufacturer’s instructions. For TPC the results were expressed as mg of gallic acid equivalents (GAE)/g of dry weight (DW) of the sample; for TFC results were expressed as mg quercetin equivalents (QE)/g of DW of the sample; for TAC determined by FRAP the results were expressed as mmol Fe^2+^ equivalents/g of DW of the sample; for TAC determined by ABTS^•+^ the results were expressed as mmol of Trolox equivalents (TE)/g of DW.

### 2.4. Quantitative Polyphenol Determination by RP-HPLC

The polyphenol analysis was carried out on a Shimadzu Prominence-i LC-2030C 3D (Shimadzu, Kyoto, Japan) system equipped with an autosampler, a binary pump, a column oven, and a photodiode array (PDA) detector. In total, 10 μL of extracts or external standards were separately injected into a C18 column (100 × 4.6 mm, 5 μm) (Thermofisher, Waltham, MA, USA) pre-heated at 30 °C. Separation was performed employing a binary mobile phase consisting of methanol (A) and water/acetic acid (97:3 % *v*/*v*) (B), at 1.2 mL/min, combined in a gradient as follows: 0 min, 95% B; 0–10 min, 95% B; 10–50 min, 95–20% B; 50–51 min, 20–0% B; 51–57 min, 0% B; 57–67 min, 0–95% B; 67–77 min, 95% B. Separations were monitored at 280 nm, and the peaks were integrated using LabSolutions software (version 5.71 SP2) (Shimadzu, Kyoto, Japan). Polyphenol identification was carried out through commercial standard injections comparing both retention times and UV spectra. Quantification was performed on an external standard method based on a calibration curve ranging from 1–50 µg/mL, with seven points for each commercial standard. The results are expressed as μg/g. Three replicates were included for each sample.

### 2.5. Dietary Fiber and Energy Determination

The soluble, insoluble, and total dietary fiber contents of all the experimental cookies and HSR were determined according to AOAC International (AOAC 991.43) through the enzymatic–gravimetric method [17]. Moisture, crude protein, crude fat, and ash were previously determined as reported in our previous paper [13]. Total carbohydrates were determined by the difference (i.e., 100 − (g [protein + fat + ash + total dietary fiber] in 100 g of DW sample)). The energy value was calculated using the Atwater factor, as follows:

Energy value (Kcal) = (%Protein × 4) + (%Fat × 9) + (%Carbohydrate × 4) + (%Total dietary fibers × 2).

### 2.6. In Vitro Upper Gastrointestinal Digestion

Simulated in vitro digestion was performed according to the INFOGEST 2.0 digestion method [18] in triplicate on all the experimental cookies. Briefly, simulated saliva fluids (SSFs), simulated gastric fluids (SGFs), and simulated intestinal fluids (SIFs) were prepared following the INFOGEST 2.0 method. Next, 5 g of each sample was mixed with 5 mL SSF containing α-amylase for 2 min at 37 °C for simulation of the oral phase. After the samples were acidified with HCl (1 M) to pH 2, 10 mL of SGF containing pepsin and gastric lipase was added. The duration of simulated gastric digestion was 2 h. After gastric digestion, the sample was neutralized to pH 7, and then 20 mL SIF containing pancreatin and bile bovine was added, and the mixture was agitated for 2 h. Heat-shock treatment was performed to inactivate the enzymes, and after cooling the mixtures were freeze-dried and stored at −80 °C until further processing.

### 2.7. Prebiotic Activity Evaluation

Evaluation of prebiotic activity was performed using *Lactobacillus rhamnosus* ATCC 7469 (LGC, Teddington, Middlesex, UK) as a probiotic bacterium that was stored by cryopreservation at −80 °C. An aliquot of stock culture was transferred aseptically to a Man, Rogosa, and Sharpe (MRS) medium plate, incubated at 37 °C for 2 days, and maintained at 4 °C [19]. The assay was performed by adding 1% (*v*/*v*) of an overnight culture of *L. rhamnosus* to separate tubes containing un-supplemented (blank) or supplemented MRS broth medium with 1% (*w*/*v*) glucose or 1% (*w*/*v*) freeze-dried in vitro digested samples (CTRc, HSc5%, and HSc10%). Cultures were incubated at 37 °C with shaking at 130 rpm for 48 h. At 0 h and 48 h samples were analyzed for enumeration in triplicate by the spread-plate method using serial dilution protocols on MRS agar plates. The plates were incubated at 37 °C for 48 h, after which the samples were enumerated as CFU/mL of culture.

### 2.8. Statistical Analysis

The mean and standard deviation (SD) of the replicates were calculated for all the analyzed data from the raw material and experimental cookie samples. Statistical analysis was performed with XLSTAT 2023.1.1 (Addinsoft SARL, New York, NY, USA) software using one-way ANOVA. Fisher’s least significant difference test was used to describe statistical differences between means at a *p* < 0.05 significance level.

## 3. Results and Discussion

### 3.1. TPC, TFC, and TAC of HS Varieties and Experimental Cookies

**Figure 1:**
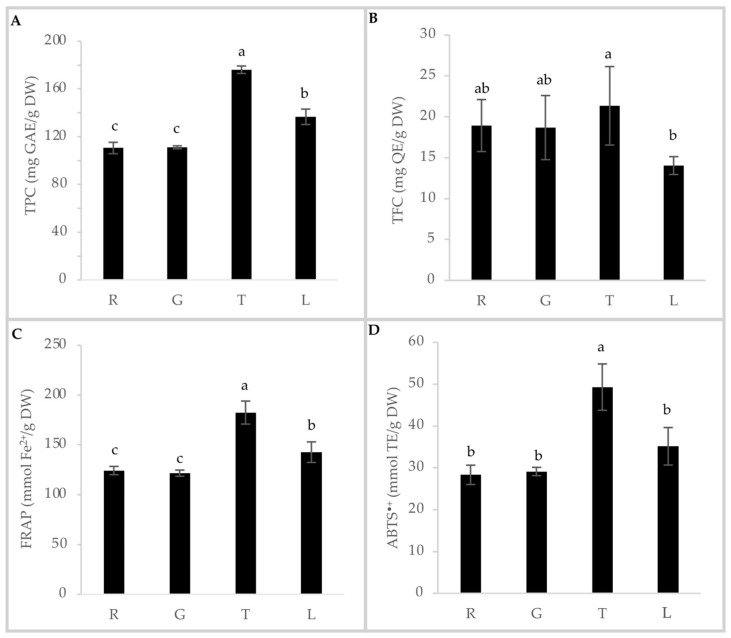
Total polyphenol content (TPC), total flavonoid content (TFC), and total antioxidant capacity (TAC) of analyzed hazelnut skins (HS) varieties. (**A**) Total phenolic content (TPC) (mg GAE/g DW); (**B**) total flavonoid content (TFC) (mg QE/g DW); (**C**) Ferric Reducing Antioxidant Power Assay (FRAP) (mmol Fe^2+^/g DW); (**D**) ABTS^•+^ radical scavenging activity (mmol TE/g DW). Data are presented as mean ± standard deviation of *n* = 3 biological replicates and *n* = 2 technical replicates. Different letters indicate significant differences (*p* ≤ 0.05), according to one-way analysis of variance. R: Tonda Gentile Romana; G: Tonda di Giffoni; L: Tonda Gentile delle Langhe; T: Turkish Tombul; GAE: Gallic Acid Equivalent; QE: Quercetin Equivalent; TE: Trolox Equivalent; DW: dry weight.

**Figure 2:**
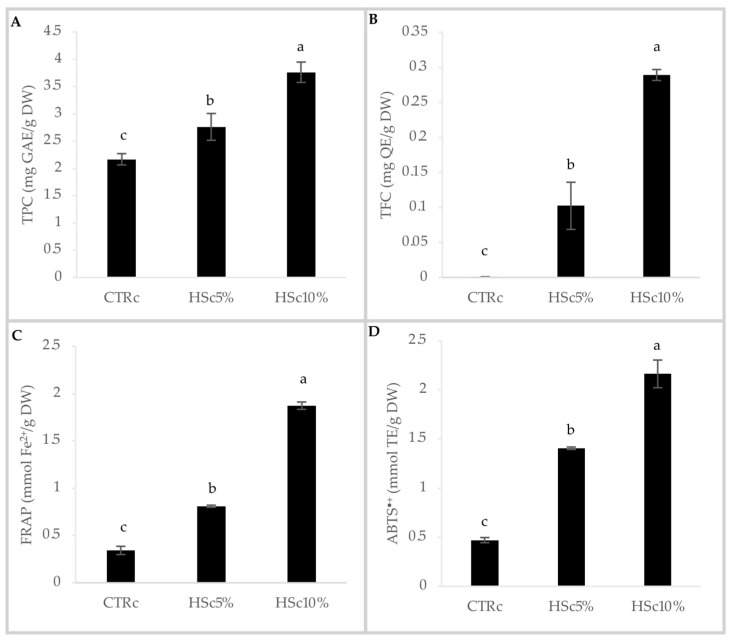
Total polyphenol content (TPC), total flavonoid content (TFC), and total antioxidant capacity (TAC) of experimental shortbread cookies. (**A**) Total phenolic content (TPC) (mg GAE/g DW); (**B**) total flavonoid content (TFC) (mg QE/g DW); (**C**) Ferric Reducing Antioxidant Power Assay (FRAP) (mmol Fe^2+^/g DW); (**D**) ABTS^•+^ radical scavenging activity (mmol TE/g DW). Data are presented as mean ± standard deviation of *n* = 3 biological replicates and *n* = 2 technical replicates. Different letters indicate significant differences (*p* ≤ 0.05), according to the one-way analysis of variance. CTRc: control cookie; HSc5%; hazelnut skin cookie 5%; HSc10%; hazelnut skin cookie 10%; GAE: Gallic Acid Equivalent; QE: Quercetin Equivalent; TE: Trolox Equivalent; DW: dry weight.

Firstly, as performed in our previous study of the nutritional profile [13], a comparison of TPC, TFC, and TAC among HSs from different hazelnut varieties was carried out, and the results are shown in Figure 1. The results for TPC (Figure 1A) and TAC from the FRAP (Figure 1C) and ABTS^•+^ assays (Figure 1D) showed a corresponding trend among the assays, with a significantly higher value for the HST variety, followed by HSL, HSR, and HSG, with the significantly lower value for TPC and FRAP, but not for ABTS^•+^, where the difference is not statistically different from HSL. The TFC analysis found less evident differences among samples, with values ranging from 13 to 22 mg QE/g of HS. It can be stated that the phenolic components and antioxidant activity determination in HS varied in the literature depending on the hazelnut varieties, the method of roasting, the applied temperatures, and the polyphenol extraction method. However, the differences among varieties followed the same trend seen in previous studies for the same type of analysis [9]. Indeed, Del Rio et al. found higher TPC and TAC/FRAP values for HST (12.7 g of polyphenols/100 g and 2206.0 mmol Fe^2+^/kg, respectively), followed by HSL (11.1 g of polyphenols/100 g and 1782.0 mmol Fe^2+^/kg, respectively), HSR (9.0 g of polyphenols/100 g and 1229.9 mmol Fe^2+^/kg, respectively), and HSG (8.7 g of polyphenols/100 g and 1321.1 mmol Fe^2+^/kg, respectively). The values found in our study were higher, which may be due to the overnight extraction time applied, compared to about one hour in the cited study [9]. However, our HST TPC results are comparable to those found by Taş and Gökmen, who applied a different extraction method [20]. As previously described [13], HSR was chosen as the best candidate for its nutritional profile and integrated into a shortbread cookie, proportionally decreasing the amount of butter in the recipe due to the presence of HSR fat and maintaining isolipidic recipes between control and experimental samples. The TPC, TFC, and TAC of the experimental cookies were also evaluated and are shown in Figure 2. The results showed that the addition of HSR to the recipe resulted in a significant and proportional increase of all the analyzed parameters, with the highest values for the HSc10% formulation (Figure 2). Our results confirmed that the addition of HS to cookies improves the antioxidant profile, as demonstrated by other authors in different food categories [21,22].

### 3.2. Quantitative Polyphenol Determination

To understand the quantitative polyphenol composition of HSR and the impact of including HSR in experimental cookies, RP-HPLC was performed, and the results are shown in Table 1. Among the compounds found in HSR were gallic acid, catechin, phloridzin, and protocatechuic acid. Gallic acid was the compound found with the highest value in HSR (398.03 µg/g), and a proportional decrease was detected in HSc10% and HSc5%, although the differences between the experimental cookies were not statistically significant. A similar trend was found for protocatechuic acid and catechin, although with lower values in comparison to gallic acid (111.63 and 257.13 µg/g for protocatechuic acid and catechin, respectively). A different trend was found for phloridzin, with values of 249.19 µg/g in HSR and a significant higher value in HSc10% in comparison to HSc5%. The exclusive presence of epicatechin gallate (3.57 µg/g) in the HSc10% cookie should be noted: this different value may be attributable to different polyphenol oxidation and degradation pathways due to high cooking temperatures [23]. The presence of gallic acid in HS as the major polyphenol compound is consistent with a previous study on HS characterization [12] and on HS-fortified foods [21], even though these results were obtained in different HS varieties (8623.0 µg/g of gallic acid in a Polish HS sample) [12].

### 3.3. Determination of Total Dietary Fibers in HSR and Experimental Cookies

Here, for the first time, the soluble, insoluble, and total dietary fiber content of hazelnut skin from the Romana variety was analyzed, and the results are shown in Table 2. The results showed that HSR had a high total fiber content of 44.13 g/100 g, most of which was insoluble dietary fiber (40.13 g/100 g). The results are comparable to those found for other Italian hazelnut varieties, although slightly lower (i.e., 54.3 g/100 g was total dietary fiber, of which 46 g/100 g was insoluble fiber, for the Tonda Gentile Trilobata and San Giovanni varieties) [22]. This is probably due to the higher content of protein (9.7 g/100 g), lipid (26.71 g/100 g), and ash (3.19 g/100 g) in HSR in comparison to other Italian HS varieties (i.e., 8.85, 17.2, and 2.5 g/100 g for protein, lipid, and ash, respectively, for Tonda Gentile Trilobata) [22]. The HS fortification of shortbread cookies resulted in a significant and proportional increase in total dietary fiber content, based on the percentage of HSR integrated into the recipe, with a higher content found for HSc10% (Table 2). The same significant difference between the experimental cookies was found for the insoluble dietary fiber content, while the difference was not significant for soluble fiber content, which, therefore, was not significantly different compared to the CTRc (Table 2).

It should be noted that, since the fiber content of HSc10% is more than 6% (i.e., 6.38 g/100 g), this food may have in Europe the nutritional claim “high fiber content” as defined by the Regulation (EC) No 1924/2006, most recently amended by Regulation (EU) No 1047/2012. Although fiber is one of the main components of HS, few works in the literature analyze fiber content following HS fortification of foods [21,22]. The results found here agree with these few studies of HS-fortified foods, although the other studies considered other HS varieties [21,22]. Therefore, the addition of HS is an excellent strategy to increase the amount of fiber in foods by starting from a waste matrix in the perspective of the circular economy.

### 3.4. Prebiotic Activity of Experimental Cookies

**Figure 3:**
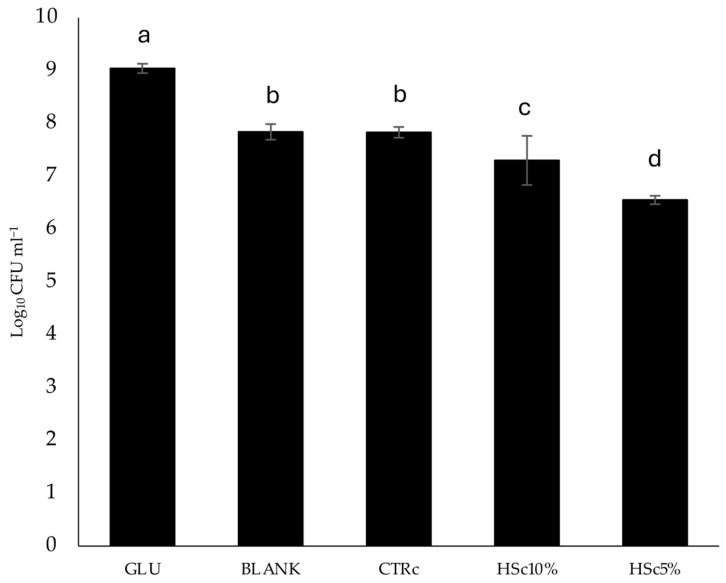
Prebiotic activity of in vitro digested experimental cookies on *L. rhamnosus*. Different letters indicate significant differences (*p* ≤ 0.05), according to the one-way analysis of variance (*n* = 3). GLU: glucose; CTRc: control cookie; HSc5%; hazelnut skin cookie 5%; HSc10%; hazelnut skin cookie 10%.

Experimental cookies were in vitro digested as described in Section 2.6, mimicking the process of upper gastrointestinal digestion. *L. rhamnosus*, a commensal bacterium of our gut microbiota, was grown on the resulting products, as described in Section 2.7, and the results are shown in Figure 3. The results showed greater colony growth for the glucose-containing control, followed by the CTRc control cookie, which was not statistically significant compared to the blank, and finally by the HSc10% and HSc5% samples, where the HSc10% sample resulted in statistically greater growth than the HSc5% sample. Although the experimental samples determine the growth of the smaller colonies, it should be considered that this in vitro procedure, although mimicking real conditions, lacked the nutrient absorption phase. Indeed, simple sugars are mainly absorbed in the small intestine [24], causing a deficiency of these simple sugars in the colon, where, indeed, commensal bacteria digest fibers that are not accessible to our enzymatic system. In consideration of this, it should be noted that, although the experimental cookies digests obtained the lowest values, the presence of greater fiber in the HSc10% formulation resulted in a significant increase in *L. rhamnosus* colonies compared to the HSc5% formulation, which could be significant in vivo compared to CTRc, and therefore in the absence of the simple sugars absorbed upstream in the small intestine. To the best of our knowledge, only two previous papers analyzed HS prebiotic activity [25,26]. Montella and colleagues found that both soluble and insoluble HS dietary fiber significantly improved the growth of *Lactobacillus plantarum* P17630 and *Lactobacillus crispatus* P1763 [25]. In their work, Alkay and co-workers found that pectin extracted from HS, after in vitro fecal fermentation, promoted *Faecalibacterium prausnitzii-* and *Lachnospiraceae*-related operational taxonomic units (OTUs) increase, whereas HS water-soluble dietary fibers and xyloglucan stimulated Bacteroides OTUs [26]. In both papers, the prebiotic activity of whole HS and HS integrated into a food, of the Tonda Gentile Romana HS variety, and toward the probiotic strain *L. rhamnosus* have never been analyzed. Taken together, these data demonstrate the potential importance of HS as a prebiotic ingredient, although further evaluations on other prebiotic strains and in vivo will be necessary to confirm these premises.

## 4. Conclusions

In addition to the previously found data, here we found that HSR resulted in a significant increase in the antioxidant capacity of experimental shortbread cookies, approximately two and five times higher in the 5% and 10% fortified recipes, respectively, than the control cookie, with gallic acid as the major antioxidant compound. For the first time, it was found that HSR had a high total fiber content of 44.13 g/100 g, most of which was insoluble dietary fiber, and so its 10% addition to the shortbread cookie recipe determined a significant increase in fiber content, making the experimental cookie earn the nutritional claim of “high fiber content” according to European regulations. Finally, preliminary in vitro evidence demonstrated that HS conferred significant prebiotic activity to a *L. rhamnosus* culture, although further confirmation of the prebiotic activity on other strains and in vivo will be necessary. Therefore, altogether the data collected for HSR could encourage companies to consider the nutritional and functional capacities of this waste matrix to create innovative supply chains that aim at the reuse of this ingredient and the creation of healthier bakery products.

## Figures and Tables

**Table 1:** Quantitative polyphenol determination in the HS of Tonda Gentile Romana and experimental cookies.

µg/g	tr (min)	HSR	HSc10%	HSc5%	CTRc
gallic acid	2.561	398.03 ± 23.62 ^a^	26.24 ± 0.05 ^b^	2.03 ± 0.02 ^b^	n.d.
protocatechuic acid	5.378	111.63 ± 8.79 ^a^	1.60 ± 0.10 ^b^	0.56 ± 0.01 ^b^	n.d.
catechin	14.525	257.13 ± 19.25 ^a^	34.96 ± 0.01 ^b^	19.96 ± 2.02 ^b^	n.d.
phloridzin	29.859	249.19 ± 17.01 ^a^	53.97 ± 7.48 ^b^	17.77 ± 0.11 ^c^	n.d.
epicatechin gallate	23.633	n.d.	3.57 ± 0.08	n.d.	n.d.

tr: retention time; HSR: hazelnut skin Romana; HSc10%: hazelnut skin cookie 10%; HSc5%: hazelnut skin cookie 5%; CTRc: control cookie; n.d.: not detected. Different letters within a row indicate significant differences (*p* ≤ 0.05), according to one-way analysis of variance (*n* = 3).

**Table 2:** Proximate composition, fiber content, and energy content of hazelnut skin of Tonda Gentile Romana and experimental cookies.

	Moisture ^4^	Protein ^1,4^	Fat ^4^	Carbohydrate ^2^	Dietary Fiber			Ash ^4^	kcal/100 g ^3^	kJ/100 g ^3^
					Total	Insoluble	Soluble			
HSR	6.31 ± 0.02 ^a^	9.70 ± 0.14 ^a^	26.71 ± 0.04 ^a^	16.28	44.13 ± 0.54 ^a^	40.13 ± 0.52 ^a^	4.00 ± 0.02 ^a^	3.19 ± 0.02 ^a^	405.23	1695.49
CTRc	4.79 ± 0.06 ^c^	7.86 ± 0.06 ^bc^	21.09 ± 0.21 ^b^	67.79	2.12 ± 1.05 ^d^	0.53 ± 0.51 ^d^	1.58 ± 0.54 ^b^	1.14 ± 0.02 ^c^	472.89	1978.59
HSc5%	3.18 ± 0.34 ^d^	7.96 ± 0.29 ^b^	21.44 ± 0.67 ^b^	65.09	4.12 ± 0.18 ^c^	2.79 ± 0.27 ^c^	1.34 ± 0.45 ^b^	1.39 ± 0.02 ^b^	477.73	1998.83
HSc10%	5.71 ± 0.04 ^b^	7.34 ± 0.23 ^c^	21.31 ± 0.46 ^b^	63.59	6.38 ± 0.56 ^b^	5.17 ± 0.85 ^b^	1.21 ± 0.29 ^b^	1.39 ± 0.01 ^b^	460.37	1926.20

Data are means ± standard deviation of two (*n* = 2) replicates. Different letters indicate significant differences (*p* ≤ 0.05), according to one-way analysis of variance. Lowercase letters represent Fisher’s test comparison among different experimental cookies. CTRc: control cookie; HSc5%: hazelnut skin cookie 5%; HSc10%: hazelnut skin cookie 10%; HSR: Romana hazelnut skin. ^1^ Conversion factor: 6.25. ^2^ As difference (i.e., 100 − (g [protein + fat + total dietary fiber + ash] in 100 g of dry weight sample)). ^3^ Per 100 g of edible part. ^4^ Data from Costantini et al., 2023 [13].

## Data Availability

The original contributions presented in this study are included in the article. Further inquiries can be directed to the corresponding author.
